# The Use of Enteral Nutrition in the Management of Stroke

**DOI:** 10.3390/nu8120827

**Published:** 2016-12-20

**Authors:** Omorogieva Ojo, Joanne Brooke

**Affiliations:** 1 Faculty of Education and Health, University of Greenwich, Avery Hill Campus, Avery Hill Road, London SE9 2UG, UK; 2 Oxford Institute of Nursing and Allied Health Research, Faculty of Health and Life Sciences, Oxford Brookes University, Oxford OX3 0FL, UK; jbrooke@brookes.ac.uk

**Keywords:** enteral nutrition, stroke, percutaneous endoscopic gastrostomy, nasogastric tube, enteral tube feeding

## Abstract

This article discusses the use of enteral nutrition in the management of stroke. Stroke is a major source of disability, including dysphagia. The clinical manifestation of swallowing difficulties in stroke patients may lead to malnutrition which has implications for health status and clinical outcomes including morbidity, mortality and cost to the health service. The prevalence of malnutrition following an acute stroke could range from 8% to 34%. Therefore, the need to develop and implement the use of enteral nutrition support in stroke patients becomes pertinent. A range of enteral feeding tubes and feeding methods may be used to support stroke patients who are unable to meet their nutritional requirements through oral intake alone, although each of these approaches has its merits and limitations. Based on this review, there is evidence that enteral nutrition support is a useful method of providing nutrition for patients with dysphagia following a stroke in order to enhance their nutritional status and promote their health. However, there are challenges in the use of enteral tube feeding in these patients.

## 1. Introduction

Enteral nutrition provides support to individuals who are unable to meet their nutritional requirements through oral intake alone. Most of these individuals have neurological conditions, such as stroke, which impact the swallowing process, causing dysphagia. 

Irrespective of the underlying medical condition, nutrition support products provide protein, energy, minerals and vitamins. These products can be given as oral nutritional supplements (ONS), when there are no contraindications, such as dysphagia, or may be delivered via enteral feeding tubes [1]. There are a range of enteral feeding tubes with different indications which can be used in the management of patients following a stroke. Short-term interventions include the insertion of a nasogastric tube (NGT), whilst long-term nutritional support involves the insertion of a percutaneous endoscopic gastrostomy (PEG) tube [2]. However, there are advantages and disadvantages to both approaches. This article discusses important aspects when planning individual care and management for patients following a stroke. 

## 2. The Consequences of Stroke

The National Institute for Health and Care Excellence (NICE) guideline [3] applied the World Health Organisation’s (WHO) definition of stroke: ‘rapidly developing clinical signs of focal (at times global) disturbance of cerebral function, lasting more than 24 h or leading to death with no apparent cause other than that of vascular origin’. Stroke has a vascular origin as it involves the disruption of blood flow to the brain, which can occur through occlusion (ischemic stroke) or rupture of a blood vessel (haemorrhagic stroke) [4]. In the UK and USA, 85% and 87%, respectively, are due to ischaemic etiology and the main risk factors include smoking and hypercholesterolemia, whereas the main risk factor for haemorrhagic stroke is hypertension [5,6,7,8]. These clinical manifestations must be distinguished from transient ischaemic attacks (TIAs) which are symptoms of stroke that resolve within 24 h and are rapidly reversible episodes of focal neurological conditions that may lead to stroke [3,9]. Globally, stroke remains the second single most common cause of death, with approximately 6.7 million deaths each year [10]. 

The consequences of stroke may be profound and can include impaired mobility, communication, dysphagia, and depression [11]. Stroke is the most common cause of acute dysphagia which may lead to malnutrition and impact on quality of life. Malnutrition immediately after stroke can reduce the chances of survival, functional ability and living conditions of residence six months later [12]. The prevalence of malnutrition following an acute stroke ranges from 8% to 34% [12]. Most patients following an acute stroke recover from dysphagia within the first four weeks, although 15% of patients may develop long-term swallowing difficulties [11,13]. Dysphagia will impact on patients’ ability to independently maintain their nutrition and hydration needs [13].

While 20% of patients following a stroke may require enteral tube feeding during the acute phase, 8% will require long-term enteral tube feeding for more than six months [14]. The provision of enteral nutrition is not without any risk of potential harm. The insertion of a PEG tube rather than a NGT is associated with less gastrointestinal bleeding and provides a higher delivery of feed [15]. Enteral feeding tubes are useful for delivering nutrition support following a stroke; however, this approach to nutrition provision impacts a patient’s quality of life, causing discomfort, restriction of movement and loss of sensation and social inclusion of eating [11]. In addition, due to the consequences of a stroke, patients who are dysphagic will have an increased risk of regurgitation and aspiration, which are complications of NGT feeding [16].

## 3. Screening for Dysphagia and Malnutrition Following an Acute Stroke

The National Institute for Health and Care Excellence (NICE) guideline [3] states that it is essential that all patients who have had an acute stroke are screened and assessed for swallowing problems by appropriately trained staff before being given any oral food, fluid or medication. The screening process of patients after stroke is often used primarily to identify those who may be at risk of aspiration and would therefore require referral for a full assessment by the speech and language therapist (SLT) who has the skills to manage dysphagia [17]. Usually, a trained nurse carries out the screening of stroke patients in acute hospital settings [17]. Patients with stroke who are not identified by the screening process to be having swallowing problems may be allowed to have food and drink orally while full assessment is being awaited [17]. Therefore, these patients may not require nutrition support or enteral tube feeding. The screening process in dysphagic stroke patients may involve a water swallow test if appropriate and is often aimed at finding a safe method to provide nutrition and hydration to these patients [13,17]. The NICE guideline [3] recommends that if the screening on admission reveals a swallowing difficulty, then the swallowing assessment should take place within 24 h following admission and should not be more than 72 h. Dysphagia includes a number of impairments that can impact swallowing which could increase the risk of malnutrition and dehydration [18]. NICE [19] also recommends screening for malnutrition and those at risk of malnutrition by healthcare professionals during the period of admission and once weekly for inpatients. It is important to consider nutrition support for people who are malnourished—those with a low body mass index (<18.5 kg/m^2^), those with unintentional weight loss (>10% within the last six months) and those who have eaten little or no food in the last five days or may eat little or no food for five days or longer [19].

Validated screening of people at risk of malnutrition involves the use of the malnutrition universal screening tool (MUST) which defines the risk of malnutrition as low risk (score 0), medium risk (score 1) and high risk (score 2 or above) [20]. This will enable early identification of those who are malnourished or at risk of malnutrition and ensure effective interventions are implemented. The assessment of patients following a stroke should include nutrition and hydration [3]. However, challenges are often encountered as patients may have difficulties communicating, and problems with mobility or standing make weight and height measurements difficult [12].

When a patient has failed a swallow test and is unable to safely tolerate oral fluids or food, an NGT should be inserted within 24 h [3,21]. If a patient is unable to tolerate a NGT, then a nasal bridle or a gastrostomy tube should be considered, along with referral to appropriate healthcare professionals for nutritional assessment, individualised care and monitoring a possible line of action [3]. It is important that healthcare professionals are aware that factors such as dysphagia, poor oral hygiene and impaired ability to self-feed may affect the nutritional status of patients following a stroke [3]. In addition, there may be no need for routine nutritional supplementation for patients who are adequately nourished [3]. However, for patients who are at risk of malnutrition, nutrition support in the form of oral nutritional supplements and/or an enteral feeding tube should be provided [3].

## 4. Enteral Nutrition in Stroke Patients

The role of nutrition support services is to treat the underlying cause of malnutrition or manage the high risk of malnutrition [22]. An integrated approach to nutrition support is essential [22]. In this regard, the services of the Home Enteral Nutrition (HEN) team, a multidisciplinary team comprising a nutrition nurse specialist, dietician and speech and language therapist, provides a useful platform for nutrition support provision in the UK [23]. 

In hospital, the nutritional status of patients following a stroke can deteriorate and malnutrition following admission has been observed to be associated with case fatality and poor functional status [24]. Decisions to commence nutrition support provision for patients following a stroke centre around the mode of delivery, whether a NGT or PEG tube, early or late initiation, duration and whether the feed should be provided during the day or overnight. In the UK, different nutrition provision regimes and approaches have been implemented across acute National Health Service (NHS) Trusts for patients following an acute stroke [24]. However, it is essential that a multidisciplinary team approach to decision-making regarding selection of a NGT or PEG tube insertion is undertaken [13,23,25].

There are medical indications for NGT and PEG tube insertions and these include people with acute stroke who are unable to tolerate adequate nutrition and fluids orally [3]. A further indication for PEG tube insertion would involve patients with stroke who are unable to swallow adequate amounts of food and fluid orally by four weeks and are at high risk of malnutrition in the long term [6,25]. However, placement of a PEG tube should be for medical reasons and not administrative convenience and each case should be considered on its merit, recognizing the clinical situation, diagnosis, prognosis and ethical issues [25]. The FOOD (Feed or Ordinary Diet) Trial Collaboration was a randomised controlled trial conducted in the UK involving dysphagic patients admitted to participating hospitals with a recent stroke [24]. Outcomes of patients with a PEG versus a NGT demonstrated a significant difference; patients with a PEG tube were more likely to have a higher mortality and poor outcomes [24]. A possible explanation is the potential impact of dependency on long-term PEG feeding as patients on PEG were still receiving feed via their PEG tube during follow-up as compared to patients on NGT [24]. In addition, the survivors in the PEG group had a lower quality of life and were more likely to be living in institutions compared with those who had NGT feeding [24].

In a recent study, ischemic stroke patients with dysphagia were compared with patients with normal swallowing ability [26]. Based on the findings, patients who had a NGT inserted were at greater risk of mortality compared to those without NGT/fed orally. A possible suggestion is that NGT insertion may place additional risk of pneumonia through colonisation of the oropharynx with pathogenic bacteria, although this position is still being debated, as the most colonisations are thought to result from bacterial origin [26].

The implication of these studies would suggest that there are merits and limitations with the use of enteral feeding tubes in dysphagic patients following a stroke. Poor outcomes reported included patients with a PEG tube being at greater risk of developing pressure sores than those with NGT, possibly due to reduced mobility and different nursing approaches [24]. An added consideration of the use of NGT in this patient cohort is the impact of the stroke on cognitive abilities and the level of confusion may lead some patients to dislodge their NGT, placing them at further risk of complications [24]. This was also confirmed by Rowat [11], who suggested no clear benefit in mortality when comparing outcomes of patients with a NGT or a PEG tube. The disadvantages of NGT for enteral nutrition remained with the risk of tube dislodgement, difficulty keeping the NGT in the stomach and the practicality of using NGT for long-term feeding [11]. However, there are other challenges with the use of PEG tubes for enteral nutrition including the risk of stoma site infection, overgranulation and buried bumper syndrome [27].

An important element of care during enteral tube feeding is the provision of regular oral hygiene, frequent replacement of the NGT and enhanced input by a physiotherapist through mobilisation of sedentary stroke patients [26]. These actions may decrease the risk of aspiration pneumonia and pressure injuries [26]. In addition, the SLT has a significant role to play in the management of dysphagia in patients with stroke. Following clinical assessment which may involve the use of modified textures of food and water, and instrumental techniques such as video-fluoroscopy, the SLT may recommend different types of exercise for the patient [13]. Based on the type of exercise therapy and its intensity, some dysphagic stroke patients may recover their swallowing and return to an oral diet [13].

Insertion of a NGT in patients following a stroke is complex due to their communication difficulties, inability to swallow and possible confusion [28]. Therefore, the use of different techniques to prevent the dislodgement of NGT is available, such as tapes, hand mittens and nasal bridles [28]. Hand mittens and nasal bridles may be effective, but informed consent and the best interest of the patient need to be carefully considered. In addition, using tape to secure the NGT to the face of the patients may not be as effective, but is a more widely accepted approach [28]. Therefore, staffs need to be trained to understand all issues regarding safe and ethical practice when securing NGTs [28].

The FOOD Trial Collaboration found a positive impact of the provision of early enteral nutrition on mortality [24]. The outcomes of this study suggested that early enteral nutrition improved mortality, but patients were more dependent on care [24]. Other studies have demonstrated the effectiveness of enteral nutrition in patients with severe stroke [29] and traumatic brain injury [30]. However, there is no current agreement among clinicians regarding the potential benefit of early commencement of NGT feeding [13].

The benefits of early initiation of enteral nutrition in patients following a stroke who are in a coma has been explored, and Yamada [31] concluded that it was nutritionally disadvantageous not to commence nutrition support within three days following admission. However, starting nutrition support too early was not nutritionally beneficial compared with early total parenteral nutrition administration, as Yamada [31] found patients were at a high risk of diarrhoea, which can predispose the patients to hypovolaemia and potentially cause ischaemia.

Diarrhoea is a common problem that is associated with enteral tube feeding and may further exacerbate the condition of patients following a stroke who are already at risk of malnutrition [32]. The duration of enteral tube feeding was associated with the risk of developing diarrhoea in patients following an acute stroke, with frequencies of between 8% and 41% [32]. The duration of enteral tube feeding of seven days or longer was associated with the occurrence of diarrhoea, whereas a duration of less than seven days did not reveal the same clinical outcome. This is an important consideration when planning and managing care after an acute stroke [32]. Furthermore, the malabsorption of enteral nutrition due to diarrhoea may lead to severe hypoproteinemia and hypoalbuminemia [31]. The mode of enteral tube feeding could be by means of bolus or continuous methods [19]. The bolus feeding method usually involves the use of a syringe to deliver feed to the patient at an agreed-upon time while the continuous feeding method requires the use of a pump and giving sets to control the rate of the administration of the feed by changing the number of drips per minute [33]. There are advantages and drawbacks in the use of these enteral feeding methods. 

## 5. Conclusions

Enteral nutrition support is a useful method of providing nutrition for patients with dysphagia following an acute stroke in order to meet their nutritional requirements. This could be delivered by means of a NGT or PEG feeding tube. Although there are advantages in these methods of enteral tube feeding, difficulties remain.
